# Crystal structure of bis­(1,10-phenanthroline-κ^2^ *N*,*N*′)(1,3-thia­zole-2-thiol­ato-κ^2^ *S* ^2^,*N*)nickel(II) hexa­fluorido­phosphate 1,4-dioxane sesquisolvate

**DOI:** 10.1107/S205698901700456X

**Published:** 2017-03-24

**Authors:** Keisuke Kai, Tomohiko Hamaguchi, Isao Ando

**Affiliations:** a Department of Chemistry, Faculty of Science, Fukuoka University, 8-19-1 Nanakuma, Jonan-ku, Fukuoka 814-0180, Japan

**Keywords:** crystal structure, 2-mercapto­thia­zolate, π–π stacking, hydrogen bonding, nickel(II) complex

## Abstract

The title salt, [Ni(C_3_H_2_NS_2_)(C_12_H_8_N_2_)_2_]PF_6_·1.5C_4_H_8_O_2_, was the unexpected product on making an attempt to prepare an [Ni(2-mercapto­thia­zol­ate)(1,10-phenanthroline)]^+^ complex by reaction of [NiCl_2_(1,10-phen­an­throline)] with 2-mercapto­thia­zolate. In the resulting complex, the 2-mercapto­thia­zolate anion acts as a chelating ligand, which coordinates to the Ni^II^ ion with the thia­zolyl N and thiol­ate S atoms. In the crystal, π–π stacking inter­actions between the coordinating 1,10-phenanthroline mol­ecules of adjacent complexes result in a zigzag chain running parallel to the *c* axis. Weak C—H⋯*X* (*X* = O, F) hydrogen-bonding inter­actions between the chains and 1,4-dioxane solvent mol­ecules and PF_6_^−^ counter-anions lead to the formation of sheets parallel to the *ac* plane.

## Abstract 2

2-Mercapto­thia­zolate is generally used as a monodentate and bridging ligand. We report here the crystal structure of a new type of nickel(II) complex in which the 2-mercapto­thia­zolate ligand acts as a chelating and non-bridging ligand.

## Chemical context   

2-Mercapto­thia­zolate (tzS) has three types of atoms available for coordination, namely the thia­zolyl N, the thia­zolyl S, and the thiol­ate S atom. Hence the tzS ligand is able to show different coordination modes. The anionic tzS ligand and its protonated neutral form are generally used as bridging ligands [*μ*_2_-tzS-*κ*(*N*, thiol­ate *S*)] or as monodentate ligands [*κ*(thiol­ate S)] (Raper *et al.*, 1989 ▸, 1990*a* ▸) whereas transition metal complexes with tzS in a bidentate coordination mode are rare (Raper *et al.*, 1989 ▸), although a number of transition metal complexes with 2-mercaptobenzo­thia­zolate as a bidentate ligand exist (Raper *et al.*, 1990*b* ▸; Ballester *et al.*, 1994 ▸; Khan *et al.*, 2010 ▸).
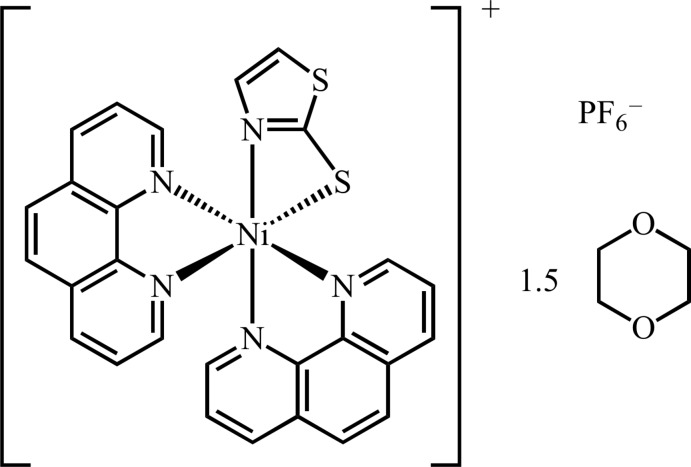


In a project intended to prepare the square-planar [Ni(tzS)(phen)]^+^ cation involving tzS as a bidentate ligand by reaction of [NiCl_2_(phen)] (phen is 1,10-phenanthroline) with 2-mercapto­thia­zolate, we obtained the unexpected title dioxane solvate compound [Ni(tzS)(phen)_2_](PF_6_)·1.5(1,4-dioxane) in which the tzS ligand acts after all as a bidentate ligand.

## Structural commentary   

**Figure 1:**
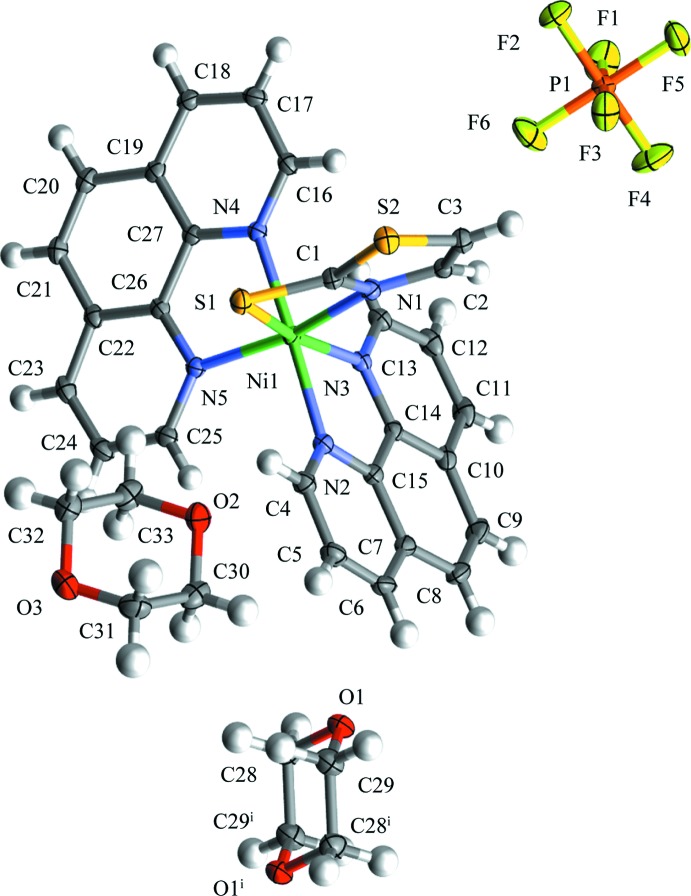
The structures of the mol­ecular entities in the title salt, shown with 50% probability displacement ellipsoids. [Symmetry code: (i) 1 − *x*, −*y*, 1 − *z*.]

The title salt consists of a complex cation [Ni(tzS)(phen)_2_]^+^, one PF_6_^−^ counter-anion, and 1.5 1,4-dioxane solvent mol­ecules of crystallization (one located about a centre of inversion), as shown in Fig. 1 ▸. The nickel(II) atom exhibits a considerably distorted octa­hedral N_5_S coordination environment, which is constructed from one bidentate tzS and two bidentate phen ligands whereby the tzS ligand chelates to the Ni^II^ atom through the thia­zolyl N and thiol­ate S atoms. Selected bond lengths and angles are gathered in Table 1 ▸. These values are very similar to that of related Ni complexes with bidentate 2-mercaptobenzo­thia­zolate ligands (Raper *et al.*, 1990*b* ▸; Ballester *et al.*, 1994 ▸; Khan *et al.*, 2010 ▸). The narrow bite angle involving the tzS ligand (Table 1 ▸) is due to formation of a four-membered chelate ring. The averaged Ni—N(phen) distances and bite angles are 2.08 Å and 80.2°, which are typical values for Ni–phen complexes (Bouzaid *et al.*, 2012 ▸).

## Supra­molecular features   

**Figure 2:**
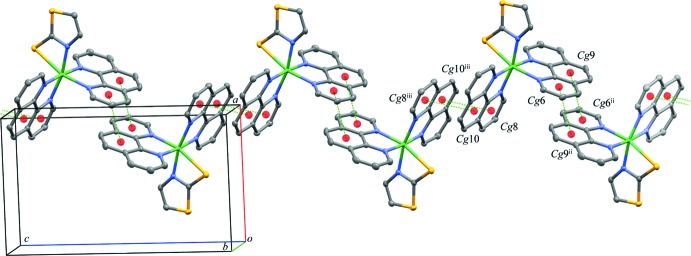
The arrangement of the complex cations in the crystal structure, forming zigzag π–π stacked chains extending parallel to the *c* axis. Green dashed lines represent π–π stacking inter­actions, red spheres represent centroids of the phen­yl/pyridyl rings. *Cg*6, *Cg*9, *Cg*8 and *Cg*10 are the centroids of the N3/C14/C10–13, C7–C10/C14/C15, N5/C26/C22–C25 and C19-C22/C26/C27 rings, respectively. [Symmetry codes: (ii) 2 − *x*, 1 − *y*, 1 − *z*; (iii) 2 − *x*, 1 − *y*, −*z*.]

**Figure 3:**
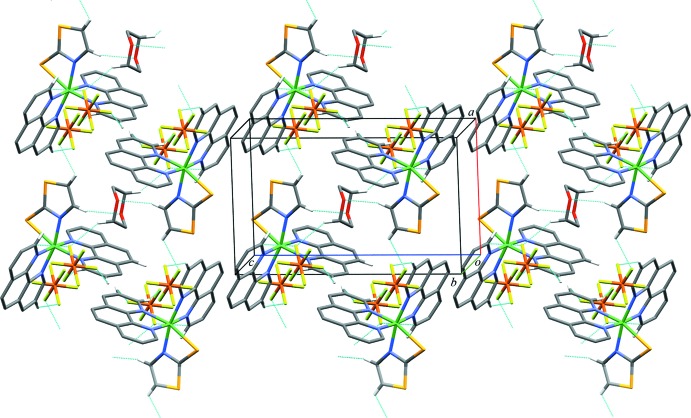
The sheet structure constructed from chains by C—H⋯*X* (*X* = F, O) hydrogen-bonding inter­actions (blue dashed lines).

In the crystal, π–π stacking inter­actions between phen ligands of adjacent [Ni(tzS)(phen)_2_]^+^ exist (Fig. 2 ▸). The inter­actions result in zigzag chains parallel to the *c* axis. The distances between the centroids of the rings are 3.8528 (11) for *Cg*6⋯*Cg*9^ii^ and *Cg*9⋯*Cg*6^ii^, and 3.6126 (10) Å for *Cg*8⋯*Cg*10^iii^ and *Cg*10⋯*Cg*8^iii^, respectively [*Cg*6, *Cg*9, *Cg*8, and *Cg*10 are the centroids of the N3/C14/C10–C13, C7–C10/C14/C15, N5/C26/C22–C25 and C19–C22/C26/C27 rings, respectively; symmetry codes: (ii) 2 − *x*, 1 − *y*, 1 − *z*; (iii) 2 − *x*, 1 − *y*, −*z*]. Such chains in turn are linked by weak C—H⋯*X* (*X* = O, F) hydrogen-bonding inter­actions involving the PF_6_^−^ counter-anion and 1,4-dioxane solvent mol­ecules, which results in the formation of a sheet structure parallel to the *ac* plane (Fig. 3 ▸, Table 2 ▸).

## Database survey   

A search in the Cambridge Structural Database (Groom *et al.*, 2016 ▸) reveals four reports of Ni complexes with bidentate 2-mercaptobenzo­thia­zolate ligands. One is a square-planar complex (Banerji *et al.*, 1982 ▸), the others being octa­hedral complexes. Two of them consist of two 2-mercaptobenzo­thia­zole ligands and another bidentate ligand (Ballester *et al.*, 1994 ▸; Khan *et al.*, 2010 ▸) whereas the third is a tris-2-mercaptobenzo­thia­zolate complex (Raper *et al.*, 1990*b* ▸). In the case of a tzS-Ni complex, one *μ*_2_-tzS-*κ*(*N*, thiol­ate *S*)-Ni_2_ complex is reported (Raper *et al.*, 1989 ▸).

## Synthesis and crystallization   

The title compound was synthesized using [NiCl_2_(phen)], prepared by a literature protocol (Yakhvarov *et al.*, 2007 ▸). A mixture of 2-mercapto­thia­zole (8.07 × 10 ^−4^ mol) and one equivalent of Et_3_N in methanol (10 ml) was added slowly to a solution of [NiCl_2_(phen)] (8.07 × 10 ^−4^ mol) in methanol (20 ml). After stirring overnight, the colour of the solution turned from blue to brown–yellow. 10 equivalents of NH_4_PF_6_ were added to the solution, resulting in a pale-brown–yellow precipitate. The precipitate was filtered off and dried *in vacuo*. The crude product containing excess NH_4_PF_6_ was purified by recrystallization using 1,4-dioxane vapor diffusion into an aceto­nitrile solution of the crude product. The title complex was isolated as brown block-like crystals [yield 365 mg, 40.6% (based on Ni)].

## Refinement   

Crystal data, data collection and structure refinement details are summarized in Table 3 ▸. H atoms were placed in calculated positions and refined as riding, with phenyl C—H = 0.95 Å and methyl­ene C—H = 0.99 Å, both with *U*_iso_(H) = 1.2*U*_eq_(C).

## Supplementary Material

Crystal structure: contains datablock(s) I. DOI: 10.1107/S205698901700456X/wm5374sup1.cif

Structure factors: contains datablock(s) I. DOI: 10.1107/S205698901700456X/wm5374Isup2.hkl

CCDC reference: 1539577

Additional supporting information:  crystallographic information; 3D view; checkCIF report

## Figures and Tables

**Table 1:** Selected geometric parameters (Å, °)

Ni1—N1	2.0524 (16)	Ni1—N2	2.0780 (15)
Ni1—N5	2.0668 (15)	Ni1—N3	2.0890 (15)
Ni1—N4	2.0735 (15)	Ni1—S1	2.5871 (5)
			
N5—Ni1—N4	80.54 (6)	N1—Ni1—S1	67.71 (4)
N2—Ni1—N3	79.90 (6)		

**Table 2:** Hydrogen-bond geometry (Å, °)

*D*—H⋯*A*	*D*—H	H⋯*A*	*D*⋯*A*	*D*—H⋯*A*
C2—H1⋯O1^i^	0.95	2.42	3.330 (2)	160
C3—H2⋯F6	0.95	2.47	3.074 (2)	122
C4—H3⋯O2	0.95	2.51	3.226 (2)	132
C5—H4⋯O1	0.95	2.63	3.269 (2)	125
C12—H9⋯F4^ii^	0.95	2.63	3.249 (3)	124
C13—H10⋯F6^ii^	0.95	2.56	3.412 (2)	150
C24—H17⋯F1^iii^	0.95	2.58	3.386 (3)	143
C28—H20⋯F3^iii^	0.99	2.50	3.336 (3)	142
C30—H23⋯F1^iv^	0.99	2.44	3.225 (3)	136

Symmetry codes: (i) 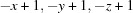; (ii) 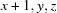; (iii) 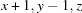; (iv) 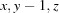.

**Table 3:** Experimental details

Crystal data	
Chemical formula	[Ni(C_3_H_2_NS_2_)(C_12_H_8_N_2_)_2_]PF_6_·1.5C_4_H_8_O_2_
*M*_r_	812.42
Crystal system, space group	Triclinic, *P* 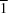
Temperature (K)	110
*a*, *b*, *c* (Å)	9.1800 (2), 12.1460 (2), 14.9005 (3)
α, β, γ (°)	88.490 (2), 89.166 (2), 83.278 (2)
*V* (Å^3^)	1649.31 (6)
*Z*	2
Radiation type	Mo *K*α
μ (mm^−1^)	0.84
Crystal size (mm)	0.27 × 0.24 × 0.16

Data collection	
Diffractometer	Rigaku Saturn 724+ CCD area-detector diffractometer
Absorption correction	Multi-scan (*CrysAlis PRO*; Rigaku Oxford Diffraction, 2015 ▸)
*T*_min_, *T*_max_	0.942, 1.000
No. of measured, independent and observed [*I* > 2σ(*I*)] reflections	30580, 9593, 8109
*R*_int_	0.037
(sin θ/λ)_max_ (Å^−1^)	0.728

Refinement	
*R*[*F* ^2^ > 2σ(*F* ^2^)], *wR*(*F* ^2^), *S*	0.041, 0.094, 1.02
No. of reflections	9593
No. of parameters	460
H-atom treatment	H-atom parameters constrained
Δρ_max_, Δρ_min_ (e Å^−3^)	1.07, −0.55

Computer programs: *CrystalClear* (Molecular Structure Corporation & Rigaku , 2001 ▸), *CrysAlis PRO* (Rigaku Oxford Diffraction, 2015 ▸), *SHELXT2014* (Sheldrick, 2015*a* ▸), *SHELXL2016* (Sheldrick, 2015*b* ▸), *Mercury* (Macrae *et al.*, 2006 ▸), *Yadokari-XG* (Kabuto *et al.*, 2009 ▸; Wakita, 2001 ▸), *PLATON* (Spek, 2009 ▸) and *publCIF* (Westrip, 2010 ▸).

## supplementary crystallographic information

### Crystal data

**Table:**

[Ni(C_3_H_2_NS_2_)(C_12_H_8_N_2_)_2_]PF_6_·1.5C_4_H_8_O_2_	*Z* = 2
*M**_r_* = 812.42	*F*(000) = 832
Triclinic, *P*1	*D*_x_ = 1.636 Mg m^−^^3^
*a* = 9.1800 (2) Å	Mo *K*α radiation, λ = 0.71073 Å
*b* = 12.1460 (2) Å	Cell parameters from 17089 reflections
*c* = 14.9005 (3) Å	θ = 2.7–31.3°
α = 88.490 (2)°	µ = 0.84 mm^−^^1^
β = 89.166 (2)°	*T* = 110 K
γ = 83.278 (2)°	Block, pale brown
*V* = 1649.31 (6) Å^3^	0.27 × 0.24 × 0.16 mm

### Data collection

**Table:**

Rigaku Saturn 724+ CCD area-detector diffractometer	8109 reflections with *I* > 2σ(*I*)
ω scans	*R*_int_ = 0.037
Absorption correction: multi-scan (*CrysAlis PRO*; Rigaku Oxford Diffraction, 2015)	θ_max_ = 31.1°, θ_min_ = 2.6°
*T*_min_ = 0.942, *T*_max_ = 1.000	*h* = −13→12
30580 measured reflections	*k* = −17→17
9593 independent reflections	*l* = −21→21

### Refinement

**Table:**

Refinement on *F*^2^	Primary atom site location: structure-invariant direct methods
Least-squares matrix: full	Secondary atom site location: difference Fourier map
*R*[*F*^2^ > 2σ(*F*^2^)] = 0.041	Hydrogen site location: inferred from neighbouring sites
*wR*(*F*^2^) = 0.094	H-atom parameters constrained
*S* = 1.02	*w* = 1/[σ^2^(*F*_o_^2^) + (0.0353*P*)^2^ + 1.5145*P*] where *P* = (*F*_o_^2^ + 2*F*_c_^2^)/3
9593 reflections	(Δ/σ)_max_ = 0.001
460 parameters	Δρ_max_ = 1.07 e Å^−^^3^
0 restraints	Δρ_min_ = −0.55 e Å^−^^3^

### Special details

**Table:**

Geometry. All esds (except the esd in the dihedral angle between two l.s. planes) are estimated using the full covariance matrix. The cell esds are taken into account individually in the estimation of esds in distances, angles and torsion angles; correlations between esds in cell parameters are only used when they are defined by crystal symmetry. An approximate (isotropic) treatment of cell esds is used for estimating esds involving l.s. planes.

### Fractional atomic coordinates and isotropic or equivalent isotropic displacement parameters (Å^2^)

**Table:**

	*x*	*y*	*z*	*U*_iso_*/*U*_eq_	
Ni1	0.73044 (2)	0.61812 (2)	0.24062 (2)	0.01241 (6)	
S1	0.53398 (5)	0.58427 (4)	0.12496 (3)	0.02027 (10)	
S2	0.27542 (5)	0.74630 (4)	0.20072 (3)	0.02077 (10)	
N1	0.53418 (17)	0.71330 (13)	0.26332 (10)	0.0156 (3)	
N2	0.67416 (17)	0.49693 (12)	0.33146 (10)	0.0149 (3)	
N3	0.83585 (16)	0.66322 (12)	0.35503 (10)	0.0141 (3)	
N4	0.80703 (16)	0.73517 (12)	0.15381 (10)	0.0135 (3)	
N5	0.91276 (17)	0.52383 (12)	0.18870 (10)	0.0145 (3)	
C1	0.4540 (2)	0.68055 (15)	0.19814 (12)	0.0168 (3)	
C2	0.4575 (2)	0.79119 (15)	0.31651 (12)	0.0163 (3)	
H1	0.500491	0.823032	0.365735	0.020*	
C3	0.3158 (2)	0.81894 (16)	0.29321 (13)	0.0200 (4)	
H2	0.248221	0.870981	0.323482	0.024*	
C4	0.5919 (2)	0.41613 (15)	0.31850 (13)	0.0193 (4)	
H3	0.556632	0.407017	0.259865	0.023*	
C5	0.5550 (2)	0.34380 (16)	0.38736 (14)	0.0233 (4)	
H4	0.496199	0.286649	0.375371	0.028*	
C6	0.6045 (2)	0.35620 (16)	0.47238 (14)	0.0235 (4)	
H5	0.579898	0.307848	0.519931	0.028*	
C7	0.6921 (2)	0.44098 (16)	0.48883 (12)	0.0191 (4)	
C8	0.7474 (2)	0.46125 (17)	0.57547 (13)	0.0249 (4)	
H6	0.726221	0.415078	0.625286	0.030*	
C9	0.8292 (2)	0.54520 (18)	0.58738 (13)	0.0254 (4)	
H7	0.864927	0.556815	0.645483	0.030*	
C10	0.8632 (2)	0.61703 (16)	0.51402 (12)	0.0191 (4)	
C11	0.9467 (2)	0.70618 (17)	0.52245 (14)	0.0239 (4)	
H8	0.984806	0.721607	0.579105	0.029*	
C12	0.9726 (2)	0.77056 (16)	0.44835 (14)	0.0222 (4)	
H9	1.029589	0.830557	0.453062	0.027*	
C13	0.9142 (2)	0.74704 (15)	0.36545 (13)	0.0178 (4)	
H10	0.931415	0.792980	0.314734	0.021*	
C14	0.8106 (2)	0.59880 (15)	0.42826 (12)	0.0151 (3)	
C15	0.7239 (2)	0.50946 (15)	0.41569 (12)	0.0156 (3)	
C16	0.7508 (2)	0.83886 (15)	0.13609 (12)	0.0169 (3)	
H11	0.667891	0.869149	0.169923	0.020*	
C17	0.8085 (2)	0.90583 (15)	0.06957 (13)	0.0194 (4)	
H12	0.764610	0.979628	0.058505	0.023*	
C18	0.9287 (2)	0.86359 (15)	0.02080 (12)	0.0179 (4)	
H13	0.968495	0.907501	−0.024998	0.021*	
C19	0.9929 (2)	0.75454 (15)	0.03913 (12)	0.0152 (3)	
C20	1.1203 (2)	0.70379 (16)	−0.00691 (12)	0.0190 (4)	
H14	1.163820	0.744055	−0.053612	0.023*	
C21	1.1801 (2)	0.59931 (16)	0.01492 (12)	0.0193 (4)	
H15	1.266761	0.568516	−0.015128	0.023*	
C22	1.1142 (2)	0.53469 (15)	0.08271 (12)	0.0161 (3)	
C23	1.1700 (2)	0.42569 (16)	0.10745 (13)	0.0202 (4)	
H16	1.258641	0.392175	0.081252	0.024*	
C24	1.0956 (2)	0.36811 (16)	0.16959 (13)	0.0210 (4)	
H17	1.131644	0.294102	0.186555	0.025*	
C25	0.9656 (2)	0.41942 (15)	0.20793 (12)	0.0178 (4)	
H18	0.913046	0.377820	0.249434	0.021*	
C26	0.98624 (19)	0.58098 (14)	0.12670 (11)	0.0140 (3)	
C27	0.92727 (19)	0.69317 (14)	0.10620 (11)	0.0134 (3)	
P1	−0.06216 (5)	1.07671 (4)	0.28379 (3)	0.01764 (10)	
F1	0.08571 (15)	1.13260 (13)	0.29517 (10)	0.0407 (4)	
F2	−0.07497 (16)	1.12468 (12)	0.18336 (8)	0.0367 (3)	
F3	−0.20929 (14)	1.02145 (11)	0.27239 (9)	0.0330 (3)	
F4	−0.04921 (18)	1.02975 (13)	0.38455 (9)	0.0462 (4)	
F5	−0.15412 (16)	1.18707 (11)	0.31890 (10)	0.0372 (3)	
F6	0.02958 (15)	0.96652 (11)	0.24816 (11)	0.0407 (4)	
O1	0.46687 (16)	0.11530 (11)	0.48337 (10)	0.0246 (3)	
C28	0.6101 (2)	0.05903 (18)	0.46710 (15)	0.0275 (4)	
H19	0.684348	0.110335	0.476684	0.033*	
H20	0.618213	0.035696	0.403887	0.033*	
C29	0.3598 (2)	0.04078 (17)	0.47172 (15)	0.0267 (4)	
H21	0.361853	0.017159	0.408583	0.032*	
H22	0.260907	0.079038	0.484938	0.032*	
O2	0.43879 (16)	0.25330 (12)	0.19127 (10)	0.0258 (3)	
C30	0.4199 (2)	0.14818 (19)	0.23091 (14)	0.0279 (5)	
H23	0.345190	0.157520	0.279569	0.034*	
H24	0.513406	0.114588	0.257485	0.034*	
C31	0.3720 (3)	0.07302 (19)	0.16157 (16)	0.0343 (5)	
H25	0.358712	0.000165	0.189903	0.041*	
H26	0.276598	0.105373	0.136657	0.041*	
O3	0.4778 (2)	0.05827 (13)	0.09123 (12)	0.0406 (4)	
C32	0.5003 (3)	0.16329 (19)	0.05165 (15)	0.0298 (5)	
H27	0.408580	0.197032	0.023020	0.036*	
H28	0.577340	0.152711	0.004431	0.036*	
C33	0.5455 (2)	0.23982 (19)	0.12063 (15)	0.0275 (4)	
H29	0.641539	0.209207	0.145690	0.033*	
H30	0.556615	0.312911	0.092026	0.033*	

### Atomic displacement parameters (Å^2^)

**Table:**

	*U*^11^	*U*^22^	*U*^33^	*U*^12^	*U*^13^	*U*^23^
Ni1	0.01357 (11)	0.01267 (11)	0.01108 (11)	−0.00176 (8)	−0.00075 (8)	−0.00039 (8)
S1	0.0239 (2)	0.0193 (2)	0.0174 (2)	−0.00050 (18)	−0.00023 (18)	−0.00451 (17)
S2	0.0155 (2)	0.0246 (2)	0.0219 (2)	−0.00020 (18)	−0.00392 (17)	−0.00288 (18)
N1	0.0160 (7)	0.0166 (7)	0.0140 (7)	−0.0014 (6)	−0.0003 (6)	0.0004 (6)
N2	0.0158 (7)	0.0137 (7)	0.0152 (7)	−0.0014 (6)	0.0007 (6)	−0.0004 (5)
N3	0.0132 (7)	0.0137 (7)	0.0153 (7)	−0.0006 (5)	−0.0018 (5)	−0.0015 (5)
N4	0.0142 (7)	0.0141 (7)	0.0124 (7)	−0.0018 (5)	−0.0019 (5)	−0.0006 (5)
N5	0.0165 (7)	0.0136 (7)	0.0133 (7)	−0.0016 (6)	−0.0016 (6)	−0.0008 (5)
C1	0.0178 (9)	0.0165 (8)	0.0159 (8)	−0.0009 (7)	−0.0001 (7)	−0.0001 (7)
C2	0.0208 (9)	0.0152 (8)	0.0134 (8)	−0.0041 (7)	0.0001 (7)	0.0006 (6)
C3	0.0205 (9)	0.0201 (9)	0.0189 (9)	0.0001 (7)	0.0019 (7)	−0.0026 (7)
C4	0.0216 (9)	0.0160 (9)	0.0209 (9)	−0.0044 (7)	−0.0007 (7)	−0.0009 (7)
C5	0.0263 (10)	0.0153 (9)	0.0288 (10)	−0.0055 (8)	0.0045 (8)	0.0001 (7)
C6	0.0289 (11)	0.0147 (9)	0.0257 (10)	−0.0004 (8)	0.0080 (8)	0.0060 (7)
C7	0.0217 (9)	0.0177 (9)	0.0163 (8)	0.0039 (7)	0.0030 (7)	0.0028 (7)
C8	0.0312 (11)	0.0260 (10)	0.0155 (9)	0.0032 (8)	0.0018 (8)	0.0054 (7)
C9	0.0310 (11)	0.0309 (11)	0.0126 (8)	0.0042 (9)	−0.0036 (8)	−0.0005 (8)
C10	0.0192 (9)	0.0211 (9)	0.0155 (8)	0.0050 (7)	−0.0034 (7)	−0.0032 (7)
C11	0.0233 (10)	0.0272 (10)	0.0206 (9)	0.0021 (8)	−0.0081 (8)	−0.0086 (8)
C12	0.0196 (9)	0.0196 (9)	0.0278 (10)	−0.0021 (7)	−0.0057 (8)	−0.0074 (8)
C13	0.0174 (9)	0.0151 (8)	0.0209 (9)	−0.0014 (7)	−0.0016 (7)	−0.0021 (7)
C14	0.0158 (8)	0.0149 (8)	0.0136 (8)	0.0032 (6)	−0.0009 (6)	−0.0022 (6)
C15	0.0171 (9)	0.0137 (8)	0.0153 (8)	0.0005 (7)	0.0008 (7)	−0.0003 (6)
C16	0.0172 (9)	0.0146 (8)	0.0184 (8)	0.0005 (7)	−0.0003 (7)	−0.0016 (7)
C17	0.0234 (10)	0.0142 (8)	0.0203 (9)	−0.0009 (7)	−0.0023 (7)	0.0020 (7)
C18	0.0219 (9)	0.0174 (9)	0.0151 (8)	−0.0052 (7)	−0.0009 (7)	0.0022 (7)
C19	0.0161 (8)	0.0177 (8)	0.0125 (8)	−0.0039 (7)	−0.0023 (6)	−0.0008 (6)
C20	0.0180 (9)	0.0244 (9)	0.0152 (8)	−0.0046 (7)	0.0023 (7)	−0.0005 (7)
C21	0.0164 (9)	0.0240 (9)	0.0173 (9)	−0.0013 (7)	0.0023 (7)	−0.0043 (7)
C22	0.0162 (8)	0.0175 (8)	0.0148 (8)	−0.0011 (7)	−0.0020 (7)	−0.0031 (6)
C23	0.0173 (9)	0.0194 (9)	0.0229 (9)	0.0036 (7)	−0.0021 (7)	−0.0061 (7)
C24	0.0235 (10)	0.0151 (8)	0.0233 (9)	0.0030 (7)	−0.0049 (8)	−0.0014 (7)
C25	0.0213 (9)	0.0154 (8)	0.0168 (8)	−0.0019 (7)	−0.0039 (7)	−0.0005 (7)
C26	0.0138 (8)	0.0142 (8)	0.0138 (8)	−0.0005 (6)	−0.0032 (6)	−0.0017 (6)
C27	0.0143 (8)	0.0142 (8)	0.0119 (7)	−0.0019 (6)	−0.0032 (6)	−0.0010 (6)
P1	0.0179 (2)	0.0176 (2)	0.0175 (2)	−0.00266 (18)	−0.00070 (18)	0.00178 (17)
F1	0.0282 (7)	0.0550 (9)	0.0433 (8)	−0.0218 (7)	0.0021 (6)	−0.0075 (7)
F2	0.0479 (9)	0.0412 (8)	0.0197 (6)	−0.0024 (7)	−0.0003 (6)	0.0089 (6)
F3	0.0254 (7)	0.0397 (8)	0.0370 (7)	−0.0140 (6)	0.0059 (5)	−0.0143 (6)
F4	0.0601 (10)	0.0546 (10)	0.0254 (7)	−0.0157 (8)	−0.0099 (7)	0.0166 (7)
F5	0.0406 (8)	0.0258 (7)	0.0450 (8)	−0.0014 (6)	0.0112 (6)	−0.0130 (6)
F6	0.0302 (7)	0.0273 (7)	0.0615 (10)	0.0095 (6)	0.0039 (7)	−0.0039 (7)
O1	0.0298 (8)	0.0153 (7)	0.0285 (8)	−0.0022 (6)	0.0013 (6)	−0.0007 (6)
C28	0.0279 (11)	0.0232 (10)	0.0314 (11)	−0.0048 (8)	0.0076 (9)	0.0008 (8)
C29	0.0253 (11)	0.0221 (10)	0.0325 (11)	−0.0013 (8)	−0.0005 (9)	−0.0006 (8)
O2	0.0232 (7)	0.0280 (8)	0.0265 (7)	−0.0036 (6)	0.0015 (6)	−0.0043 (6)
C30	0.0207 (10)	0.0383 (12)	0.0241 (10)	−0.0018 (9)	−0.0007 (8)	0.0059 (9)
C31	0.0444 (14)	0.0247 (11)	0.0345 (12)	−0.0083 (10)	0.0017 (10)	0.0040 (9)
O3	0.0588 (12)	0.0220 (8)	0.0392 (10)	0.0015 (8)	0.0122 (8)	−0.0051 (7)
C32	0.0342 (12)	0.0316 (12)	0.0231 (10)	−0.0022 (9)	0.0043 (9)	−0.0001 (9)
C33	0.0198 (10)	0.0352 (12)	0.0279 (11)	−0.0059 (9)	0.0011 (8)	0.0020 (9)

### Geometric parameters (Å, º)

**Table:**

Ni1—N1	2.0524 (16)	C17—H12	0.9500
Ni1—N5	2.0668 (15)	C18—C19	1.407 (3)
Ni1—N4	2.0735 (15)	C18—H13	0.9500
Ni1—N2	2.0780 (15)	C19—C27	1.402 (2)
Ni1—N3	2.0890 (15)	C19—C20	1.432 (3)
Ni1—S1	2.5871 (5)	C20—C21	1.355 (3)
S1—C1	1.7143 (19)	C20—H14	0.9500
S2—C3	1.724 (2)	C21—C22	1.433 (3)
S2—C1	1.7377 (19)	C21—H15	0.9500
N1—C1	1.324 (2)	C22—C26	1.403 (2)
N1—C2	1.373 (2)	C22—C23	1.404 (3)
N2—C4	1.326 (2)	C23—C24	1.368 (3)
N2—C15	1.360 (2)	C23—H16	0.9500
N3—C13	1.327 (2)	C24—C25	1.401 (3)
N3—C14	1.359 (2)	C24—H17	0.9500
N4—C16	1.325 (2)	C25—H18	0.9500
N4—C27	1.359 (2)	C26—C27	1.433 (2)
N5—C25	1.329 (2)	P1—F3	1.5901 (13)
N5—C26	1.359 (2)	P1—F2	1.5925 (13)
C2—C3	1.353 (3)	P1—F4	1.5929 (14)
C2—H1	0.9500	P1—F5	1.5944 (14)
C3—H2	0.9500	P1—F6	1.5946 (14)
C4—C5	1.397 (3)	P1—F1	1.6002 (14)
C4—H3	0.9500	O1—C29	1.427 (2)
C5—C6	1.370 (3)	O1—C28	1.430 (3)
C5—H4	0.9500	C28—C29^i^	1.499 (3)
C6—C7	1.407 (3)	C28—H19	0.9900
C6—H5	0.9500	C28—H20	0.9900
C7—C15	1.402 (2)	C29—H21	0.9900
C7—C8	1.431 (3)	C29—H22	0.9900
C8—C9	1.352 (3)	O2—C30	1.421 (3)
C8—H6	0.9500	O2—C33	1.427 (3)
C9—C10	1.434 (3)	C30—C31	1.500 (3)
C9—H7	0.9500	C30—H23	0.9900
C10—C14	1.405 (2)	C30—H24	0.9900
C10—C11	1.407 (3)	C31—O3	1.419 (3)
C11—C12	1.370 (3)	C31—H25	0.9900
C11—H8	0.9500	C31—H26	0.9900
C12—C13	1.403 (3)	O3—C32	1.427 (3)
C12—H9	0.9500	C32—C33	1.498 (3)
C13—H10	0.9500	C32—H27	0.9900
C14—C15	1.436 (2)	C32—H28	0.9900
C16—C17	1.403 (3)	C33—H29	0.9900
C16—H11	0.9500	C33—H30	0.9900
C17—C18	1.369 (3)		
			
N1—Ni1—N5	167.32 (6)	C17—C18—H13	120.3
N1—Ni1—N4	93.43 (6)	C19—C18—H13	120.3
N5—Ni1—N4	80.54 (6)	C27—C19—C18	117.60 (17)
N1—Ni1—N2	91.11 (6)	C27—C19—C20	118.87 (17)
N5—Ni1—N2	95.58 (6)	C18—C19—C20	123.53 (17)
N4—Ni1—N2	174.56 (6)	C21—C20—C19	121.19 (17)
N1—Ni1—N3	96.71 (6)	C21—C20—H14	119.4
N5—Ni1—N3	95.07 (6)	C19—C20—H14	119.4
N4—Ni1—N3	96.57 (6)	C20—C21—C22	120.89 (17)
N2—Ni1—N3	79.90 (6)	C20—C21—H15	119.6
N1—Ni1—S1	67.71 (4)	C22—C21—H15	119.6
N5—Ni1—S1	100.94 (4)	C26—C22—C23	117.41 (17)
N4—Ni1—S1	89.91 (4)	C26—C22—C21	119.09 (17)
N2—Ni1—S1	94.59 (4)	C23—C22—C21	123.49 (17)
N3—Ni1—S1	163.53 (4)	C24—C23—C22	119.49 (18)
C1—S1—Ni1	72.66 (6)	C24—C23—H16	120.3
C3—S2—C1	90.25 (9)	C22—C23—H16	120.3
C1—N1—C2	112.89 (16)	C23—C24—C25	119.32 (18)
C1—N1—Ni1	100.84 (12)	C23—C24—H17	120.3
C2—N1—Ni1	146.25 (13)	C25—C24—H17	120.3
C4—N2—C15	118.14 (16)	N5—C25—C24	122.78 (18)
C4—N2—Ni1	128.68 (13)	N5—C25—H18	118.6
C15—N2—Ni1	113.07 (12)	C24—C25—H18	118.6
C13—N3—C14	118.13 (16)	N5—C26—C22	122.98 (16)
C13—N3—Ni1	129.09 (13)	N5—C26—C27	117.26 (16)
C14—N3—Ni1	112.67 (11)	C22—C26—C27	119.76 (16)
C16—N4—C27	118.16 (15)	N4—C27—C19	122.76 (16)
C16—N4—Ni1	129.33 (13)	N4—C27—C26	117.15 (15)
C27—N4—Ni1	112.38 (11)	C19—C27—C26	120.09 (16)
C25—N5—C26	117.93 (16)	F3—P1—F2	90.17 (8)
C25—N5—Ni1	129.51 (13)	F3—P1—F4	90.13 (8)
C26—N5—Ni1	112.56 (11)	F2—P1—F4	179.51 (9)
N1—C1—S1	118.78 (14)	F3—P1—F5	90.39 (8)
N1—C1—S2	111.99 (14)	F2—P1—F5	90.13 (8)
S1—C1—S2	129.24 (11)	F4—P1—F5	89.48 (9)
C3—C2—N1	114.64 (17)	F3—P1—F6	89.55 (8)
C3—C2—H1	122.7	F2—P1—F6	89.58 (8)
N1—C2—H1	122.7	F4—P1—F6	90.81 (9)
C2—C3—S2	110.23 (14)	F5—P1—F6	179.70 (9)
C2—C3—H2	124.9	F3—P1—F1	179.86 (10)
S2—C3—H2	124.9	F2—P1—F1	89.74 (8)
N2—C4—C5	122.83 (18)	F4—P1—F1	89.96 (8)
N2—C4—H3	118.6	F5—P1—F1	89.50 (8)
C5—C4—H3	118.6	F6—P1—F1	90.56 (8)
C6—C5—C4	119.28 (18)	C29—O1—C28	109.56 (15)
C6—C5—H4	120.4	O1—C28—C29^i^	111.04 (17)
C4—C5—H4	120.4	O1—C28—H19	109.4
C5—C6—C7	119.59 (18)	C29^i^—C28—H19	109.4
C5—C6—H5	120.2	O1—C28—H20	109.4
C7—C6—H5	120.2	C29^i^—C28—H20	109.4
C15—C7—C6	117.20 (18)	H19—C28—H20	108.0
C15—C7—C8	119.17 (18)	O1—C29—C28^i^	110.46 (18)
C6—C7—C8	123.63 (18)	O1—C29—H21	109.6
C9—C8—C7	120.92 (18)	C28^i^—C29—H21	109.6
C9—C8—H6	119.5	O1—C29—H22	109.6
C7—C8—H6	119.5	C28^i^—C29—H22	109.6
C8—C9—C10	121.29 (18)	H21—C29—H22	108.1
C8—C9—H7	119.4	C30—O2—C33	109.66 (16)
C10—C9—H7	119.4	O2—C30—C31	110.18 (17)
C14—C10—C11	117.21 (18)	O2—C30—H23	109.6
C14—C10—C9	118.94 (18)	C31—C30—H23	109.6
C11—C10—C9	123.85 (18)	O2—C30—H24	109.6
C12—C11—C10	119.52 (18)	C31—C30—H24	109.6
C12—C11—H8	120.2	H23—C30—H24	108.1
C10—C11—H8	120.2	O3—C31—C30	110.5 (2)
C11—C12—C13	119.36 (18)	O3—C31—H25	109.5
C11—C12—H9	120.3	C30—C31—H25	109.5
C13—C12—H9	120.3	O3—C31—H26	109.5
N3—C13—C12	122.67 (18)	C30—C31—H26	109.5
N3—C13—H10	118.7	H25—C31—H26	108.1
C12—C13—H10	118.7	C31—O3—C32	109.84 (17)
N3—C14—C10	123.10 (17)	O3—C32—C33	111.09 (18)
N3—C14—C15	117.20 (15)	O3—C32—H27	109.4
C10—C14—C15	119.70 (17)	C33—C32—H27	109.4
N2—C15—C7	122.97 (17)	O3—C32—H28	109.4
N2—C15—C14	117.04 (16)	C33—C32—H28	109.4
C7—C15—C14	119.99 (17)	H27—C32—H28	108.0
N4—C16—C17	122.88 (17)	O2—C33—C32	110.56 (17)
N4—C16—H11	118.6	O2—C33—H29	109.5
C17—C16—H11	118.6	C32—C33—H29	109.5
C18—C17—C16	119.20 (17)	O2—C33—H30	109.5
C18—C17—H12	120.4	C32—C33—H30	109.5
C16—C17—H12	120.4	H29—C33—H30	108.1
C17—C18—C19	119.37 (17)		
			
C2—N1—C1—S1	179.41 (13)	N3—C14—C15—C7	−179.09 (16)
Ni1—N1—C1—S1	−1.39 (16)	C10—C14—C15—C7	0.0 (3)
C2—N1—C1—S2	−0.3 (2)	C27—N4—C16—C17	1.2 (3)
Ni1—N1—C1—S2	178.89 (9)	Ni1—N4—C16—C17	−174.37 (14)
Ni1—S1—C1—N1	1.14 (13)	N4—C16—C17—C18	−0.5 (3)
Ni1—S1—C1—S2	−179.20 (15)	C16—C17—C18—C19	−0.8 (3)
C3—S2—C1—N1	0.03 (15)	C17—C18—C19—C27	1.3 (3)
C3—S2—C1—S1	−179.65 (14)	C17—C18—C19—C20	−178.73 (18)
C1—N1—C2—C3	0.5 (2)	C27—C19—C20—C21	−1.8 (3)
Ni1—N1—C2—C3	−178.06 (17)	C18—C19—C20—C21	178.29 (18)
N1—C2—C3—S2	−0.5 (2)	C19—C20—C21—C22	2.3 (3)
C1—S2—C3—C2	0.25 (15)	C20—C21—C22—C26	0.0 (3)
C15—N2—C4—C5	−0.1 (3)	C20—C21—C22—C23	179.21 (18)
Ni1—N2—C4—C5	−175.92 (14)	C26—C22—C23—C24	2.7 (3)
N2—C4—C5—C6	0.3 (3)	C21—C22—C23—C24	−176.58 (18)
C4—C5—C6—C7	−0.3 (3)	C22—C23—C24—C25	−0.5 (3)
C5—C6—C7—C15	0.2 (3)	C26—N5—C25—C24	2.4 (3)
C5—C6—C7—C8	179.18 (19)	Ni1—N5—C25—C24	−176.95 (13)
C15—C7—C8—C9	0.0 (3)	C23—C24—C25—N5	−2.2 (3)
C6—C7—C8—C9	−179.0 (2)	C25—N5—C26—C22	0.0 (3)
C7—C8—C9—C10	0.2 (3)	Ni1—N5—C26—C22	179.44 (13)
C8—C9—C10—C14	−0.4 (3)	C25—N5—C26—C27	179.54 (15)
C8—C9—C10—C11	179.5 (2)	Ni1—N5—C26—C27	−1.01 (19)
C14—C10—C11—C12	0.2 (3)	C23—C22—C26—N5	−2.5 (3)
C9—C10—C11—C12	−179.65 (19)	C21—C22—C26—N5	176.79 (16)
C10—C11—C12—C13	0.6 (3)	C23—C22—C26—C27	177.96 (16)
C14—N3—C13—C12	0.7 (3)	C21—C22—C26—C27	−2.8 (3)
Ni1—N3—C13—C12	176.59 (14)	C16—N4—C27—C19	−0.5 (2)
C11—C12—C13—N3	−1.1 (3)	Ni1—N4—C27—C19	175.75 (13)
C13—N3—C14—C10	0.1 (3)	C16—N4—C27—C26	179.86 (15)
Ni1—N3—C14—C10	−176.42 (14)	Ni1—N4—C27—C26	−3.88 (19)
C13—N3—C14—C15	179.19 (16)	C18—C19—C27—N4	−0.7 (3)
Ni1—N3—C14—C15	2.7 (2)	C20—C19—C27—N4	179.34 (16)
C11—C10—C14—N3	−0.5 (3)	C18—C19—C27—C26	178.90 (16)
C9—C10—C14—N3	179.28 (17)	C20—C19—C27—C26	−1.0 (2)
C11—C10—C14—C15	−179.60 (17)	N5—C26—C27—N4	3.4 (2)
C9—C10—C14—C15	0.2 (3)	C22—C26—C27—N4	−177.08 (15)
C4—N2—C15—C7	0.0 (3)	N5—C26—C27—C19	−176.28 (15)
Ni1—N2—C15—C7	176.42 (14)	C22—C26—C27—C19	3.3 (2)
C4—N2—C15—C14	−178.94 (16)	C29—O1—C28—C29^i^	−57.7 (2)
Ni1—N2—C15—C14	−2.5 (2)	C28—O1—C29—C28^i^	57.4 (2)
C6—C7—C15—N2	0.0 (3)	C33—O2—C30—C31	−58.8 (2)
C8—C7—C15—N2	−179.08 (17)	O2—C30—C31—O3	59.4 (2)
C6—C7—C15—C14	178.90 (17)	C30—C31—O3—C32	−57.7 (3)
C8—C7—C15—C14	−0.2 (3)	C31—O3—C32—C33	56.7 (3)
N3—C14—C15—N2	−0.1 (2)	C30—O2—C33—C32	57.7 (2)
C10—C14—C15—N2	179.01 (16)	O3—C32—C33—O2	−57.0 (2)

Symmetry code: (i) −*x*+1, −*y*, −*z*+1.

### Hydrogen-bond geometry (Å, º)

**Table:**

*D*—H···*A*	*D*—H	H···*A*	*D*···*A*	*D*—H···*A*
C2—H1···O1^ii^	0.95	2.42	3.330 (2)	160
C3—H2···F6	0.95	2.47	3.074 (2)	122
C4—H3···O2	0.95	2.51	3.226 (2)	132
C5—H4···O1	0.95	2.63	3.269 (2)	125
C12—H9···F4^iii^	0.95	2.63	3.249 (3)	124
C13—H10···F6^iii^	0.95	2.56	3.412 (2)	150
C24—H17···F1^iv^	0.95	2.58	3.386 (3)	143
C28—H20···F3^iv^	0.99	2.50	3.336 (3)	142
C30—H23···F1^v^	0.99	2.44	3.225 (3)	136

Symmetry codes: (ii) −*x*+1, −*y*+1, −*z*+1; (iii) *x*+1, *y*, *z*; (iv) *x*+1, *y*−1, *z*; (v) *x*, *y*−1, *z*.
